# A 45-Second Self-Test for Cardiorespiratory Fitness: Heart Rate-Based Estimation in Healthy Individuals

**DOI:** 10.1371/journal.pone.0168154

**Published:** 2016-12-13

**Authors:** Francesco Sartor, Matteo Bonato, Gabriele Papini, Andrea Bosio, Rahil A. Mohammed, Alberto G. Bonomi, Jonathan P. Moore, Giampiero Merati, Antonio La Torre, Hans-Peter Kubis

**Affiliations:** 1 Personal Health, Philips Research, Eindhoven, The Netherlands; 2 Department of Biomedical Sciences for Health, Università degli Studi di Milano, Milan, Italy; 3 Department of Information Engineering, University of Pisa, Pisa, Italy; 4 Department of Electrical Engineering, Eindhoven University of Technology, Eindhoven, The Netherlands; 5 Mapei Sport, Olgiate Olona (Varese), Italy; 6 College of Health and Behavioural Sciences, Bangor University, Bangor, United Kingdom; 7 School of Physical Education, University of Sulaimani, Sulaimani, Iraq; Kurume University School of Medicine, JAPAN

## Abstract

Cardio-respiratory fitness (CRF) is a widespread essential indicator in Sports Science as well as in Sports Medicine. This study aimed to develop and validate a prediction model for CRF based on a 45 second self-test, which can be conducted anywhere. Criterion validity, test re-test study was set up to accomplish our objectives. Data from 81 healthy volunteers (age: 29 ± 8 years, BMI: 24.0 ± 2.9), 18 of whom females, were used to validate this test against gold standard. Nineteen volunteers repeated this test twice in order to evaluate its repeatability. CRF estimation models were developed using heart rate (HR) features extracted from the resting, exercise, and the recovery phase. The most predictive HR feature was the intercept of the linear equation fitting the HR values during the recovery phase normalized for the height^2^ (r^2^ = 0.30). The Ruffier-Dickson Index (RDI), which was originally developed for this squat test, showed a negative significant correlation with CRF (r = -0.40), but explained only 15% of the variability in CRF. A multivariate model based on RDI and sex, age and height increased the explained variability up to 53% with a cross validation (CV) error of 0.532 L ∙ min^-1^ and substantial repeatability (ICC = 0.91). The best predictive multivariate model made use of the linear intercept of HR at the beginning of the recovery normalized for height^2^ and age^2^; this had an adjusted r^2^ = 0. 59, a CV error of 0.495 L·min^-1^ and substantial repeatability (ICC = 0.93). It also had a higher agreement in classifying CRF levels (κ = 0.42) than RDI-based model (κ = 0.29). In conclusion, this simple 45 s self-test can be used to estimate and classify CRF in healthy individuals with moderate accuracy and large repeatability when HR recovery features are included.

## Introduction

Maximal oxygen uptake (${\dot{\text{V}}}_{\text{O}2\text{max}}$) estimation is essential in sports, clinical and home settings [1] because it is considered the best index of cardio-respiratory fitness (CRF) [2], which expresses the maximal aerobic power of an individual [3]. The importance of ${\dot{\text{V}}}_{\text{O}2\text{max}}$ as predictor of cardiovascular disease and mortality has been recently highlighted by a scientific statement issued by the American Heart Association [4]. Remarkably, individuals with low cardiorespiratory fitness had a 70% higher risk of all cause-mortality and a 56% risk of cardiovascular disease mortality [4]. The inclusion of cardio-respiratory fitness in the traditional cardiovascular disease risk score is currently being evaluated [5]. Nevertheless, the direct assessment of ${\dot{\text{V}}}_{\text{O}2\text{max}}$ implies a higher risk of adverse cardiovascular events, costly equipment for the measurement of the expired gases, and trained personnel. Moreover, lack of motivation, general discomfort, reduced exercise tolerance, and the occurrence of symptoms can hamper its correct assessment [2]. Maximal exercise criteria are often unmet in non-athlete populations [6, 7]. For these reasons a large number of submaximal protocols have been designed to estimate ${\dot{\text{V}}}_{\text{O}2\text{max}}$. We have recently reviewed submaximal exercise protocols employing different modalities such as cycling, walking, running, treadmill, stepping and many others, which can be performed in laboratories as well as in health/fitness centres or at school [1]. However, most of them require some sort of equipment, a large space, and a 3 to 6 minute exercise protocol. Only a few can be executed anywhere and in a short period of time. We have identified the 45 s squat test, also known as the Ruffier-Dickson test, as a suitable protocol to provide an easy and fast self-evaluation of ${\dot{\text{V}}}_{\text{O}2\text{max}}$.

The Ruffier-Dickson test was originally designed by J.E. Ruffier and modified by J. Dickson, who developed the Ruffier-Dickson index (RDI), and it has been used to classify cardio-respiratory fitness [8]. However, the only data available to compare ${\dot{\text{V}}}_{\text{O}2\text{max}}$ with RDI was published in Rugby players [9]. Using those rugby players data we have shown a moderately valid model for estimating ${\dot{\text{V}}}_{\text{O}2\text{max}}$ in these players (see Sartor et al. [1]). Nevertheless, this model was developed in a specific population of athletes with a reasonably high ${\dot{\text{V}}}_{\text{O}2\text{max}}$, it was not cross validated, and it was based on a small sample size of 22 players [1, 9]. Furthermore, to our knowledge there is no evidence to judge how well the CRF levels are classified by the RDI, although this was one of the main reasons this index was designed for.

The aim of the present study was to validate as well as cross-validate the 45-second squat test in a larger population with a wider range of ${\dot{\text{V}}}_{\text{O}2\text{max}}$. The secondary objective was to evaluate the correlation between the original Ruffier-Dickson index (RDI) and ${\dot{\text{V}}}_{\text{O}2\text{max}}$, and its CRF classification value. Moreover, the repeatability of the model developed was evaluated via a test re-test design.

## Materials and Methods

### Participants

This study has been conducted in four different centres: Bangor University in the United Kingdom, Philips Research in The Netherlands, and Università degli Studi di Milano (Milan, Italy), and Mapei-Sport in Italy. The volunteers were recruited via posters and newsletters. Volunteers were excluded if they suffered from any chronic disease; if they had functional or cognitive impediments; or if they were taking any medication affecting the hormonal and metabolic systems. In total 94 healthy volunteers were included in the study data from 13 subjects were excluded because of incompleteness or technical difficulties (e.g. HR data were too noisy for various reasons and could not be improved by low-pass filtering). The characteristics of the remaining 81 subjects are reported in Table 1. The study protocol was reviewed and approved by the Departmental Ethics Committees of Bangor’s School of Sport Health and Exercise Sciences, the Internal Committee of Biomedical Experiments of Philips Research and Institutional Ethics Review Committee of the Università degli Studi di Milano in conformity with the Declaration of Helsinki. All subjects have signed the written informed consent prior to testing.

**Table 1:** Subjects characteristics.

	Males	Females	All
	Means ± SDs (Min ÷ Max)	Means ± SDs (Min ÷ Max)	Means ± SDs
	n = 63	n = 18	n = 81
Age (years)	29 ± 8 (18 ÷ 67)	27 ± 9 (19 ÷ 46)	29 ± 8
Height (m)	1.76 ± 0.06 (1.66 ÷ 1.88)	1.70 ± 0.05 (1.61 ÷ 1.76)	1.74 ± 0.06
Weight (kg)	74.5 ± 9.8 (58 ÷ 100)	68.0 ± 10.7 (52.5 ÷ 86.95)	73.1 ± 10.3
BMI	24.1 ± 2.9 (18.8 ÷ 33.6)	23.5 ± 3.0 (19.0 ÷ 29.22)	24.0 ± 2.9
HR_rest_ (beats·min^-1^)	66 ± 10 (46 ÷ 93)	70 ± 11 (47 ÷ 96)	67 ± 11
${\dot{\text{V}}}_{\text{O}2\text{max}}$ (L·min^-1^)	3.15 ± 0.72 (1.96 ÷ 5.22)	2.27 ± 0.47 (1.35 ÷ 3.18)	2.95 ± 0.77
${\dot{\text{V}}}_{\text{O}2\text{max}}$ (mL·kg^-1^·min^-1^)	43.4 ± 11.7 (27.0 ÷ 77.8)	33.8 ± 6.9 (23.7 ÷ 43.4)	41.3 ± 11.5
HR_max_ (beats·min^-1^)	187 ± 10 (150 ÷ 209)	184 ± 13 (157 ÷ 202)	186 ± 11

### Submaximal and maximal cardiorespiratory fitness assessment

Bare-footed subjects ‘stature was measured using a wall-mounted stadiometer (Bodycare Products, Southam, United Kingdom, used in Bangor, Seca 217, Vogel & Halke, Hamburg, Germany, used in Eindhoven, Varese, and Milan). A digital scale (Seca; Vogel & Halke, Hamburg, Germany, used in Bangor and Milan, BC-533, Tanita, Amsterdam, the Netherlands, used in Eindhoven, RB-L 200, and Wunder, Trezzo sull’Adda (MI), Italy, used inVarese) was used to measure body weight of subjects in their underwear.

Subjects lied down (supine) for 5 minutes in order to rest. At the end of the 5 minutes rest subjects stood up. The researcher monitored the HR until the HR stabilized in the standing position. Once the HR reached steady state in the standing position (this usually required only a few seconds), subjects started squatting. Subjects performed 30 squats in 45 s, following the tempo set by a metronome (80 beats·min^-1^), executing a down and up movement. The squatting movement consisted of flexion of the knees to 90°, keeping the back straight and the arms extended frontally. Complete squatting was avoided in order to make this test feasible to a large range of people. At the end of the 45 seconds the subjects lied down (supine) and recovered for 3 minutes. Heart rate was recorded in inter-beat-interval mode throughout the entire test including the resting and recovery phases by means of a chest strap HR monitor (Polar, RS800CX; Polar Electro, Kempele, Finland). Nineteen non-athlete subjects repeated the squat test on a different day at least one week after the first test.

A few minutes after having completed the Ruffier-Dickson squat test when HR was ≤ initial resting HR (and in any case not earlier than 10 min) the subjects moved to the stationary cycle ergometer (Lode Excalibur Sport PFM, Groningen, The Netherlands, used in Bangor, Eindhoven and Varese; and a Monark Ergomedic 839 E, Monark, Varberg, Sweden, used in Milan) and were prepared for the incremental test to exhaustion to determine their maximum oxygen uptake. Prior to the test, the subjects warmed-up for 10 min on the cycle ergometer with no resistance at a pedalling rate between 90 and 100 rpm. The incremental exercise began at a workload of 75 W for 3 min to increase by 25 W every minute until volitional exhaustion, which was defined as the inability to maintain a cadence of 90 rpm for more than 5 s despite strong verbal encouragement. During the test, ${\dot{\text{V}}}_{\text{O}2}$, ${\dot{\text{V}}}_{\text{CO}2}$, ventilation and respiratory rate were continuously monitored breath by breath (MetaLyzer 3B, Leipzig, Germany, used in Bangor; Oxycon Pro Metabolic Cart, Carefusion, California, USA, used in Eindhoven; Vmax29, Sensormedics, Yorba Linda, CA, USA, used in Varese; and Cosmed Quark CPET, Cosmed, Roma, Italy, used in Milan,). HR was measured continuously using the same heart rate monitor worn during the Ruffier-Dickson squatting test.

In addition to the 94 healthy volunteers tested to validate the squat test 10 more subjects (age = 24 ± 5, BMI = 23.5 ± 4.1, 60% males) were recruited in order to evaluate the aerobic component of the 45 seconds squat test. These subjects were asked to perform the same test while wearing a breath by breath indirect calorimeter (k4b2, Cosmed, Albano Laziale, Italy), calibrated before each test according to the maker’s requirements.

### Multiple regression models

All data collected in this study were processed by using Matlab software (R2013b, Matworks). The raw beat-to-beat HR intervals, in milliseconds, were converted into HR, in beats·min^-1^, which was re-sampled to 1 Hz. Peaks were removed according with the flowing logic operation. For each HR sample the short term variation, defined as the standard deviation in an interval of 3 samples, was compared to the long term variation, defined as the standard deviation in an interval of 21 samples. When the short term standard deviation was at least 10 times greater than the long term standard deviation, the HR sample was replaced with the average of the previous and following HR samples. Furthermore, the signal was low-pass filtered using a 5 samples moving average. Each signal was visually inspected to ensure that this cleaning procedure did not alter the morphology of the signal. A total of 25 features were extracted from the HR signal during the resting, squatting and recovery phase. These HR features together with subjects’ characteristics (e.g. age, height and weight, etc) were used to build the prediction models. The following HR features were considered. Rest HR mode was defined as the mode HR value during 5 min of resting in the supine position, excluding the first and the last minute of measurement. Squat peak was the maximum HR observed during the squatting exercise. The recovery HR linear intercept (named for simplicity here, start HR_rec_) was the intercept (b) of the linear fit y = m x + b (where m was the angular coefficient of the linear fit) of the HR signal corresponding to the complete 180 s recovery phase. Recovery exponential coefficient was the coefficient (d) of the exponential fit (y = d · e^fx^) of the HR decay during the recovery phase. Recovery HR end was the final value of the complete recovery phase. Finally RDI was calculated as previously described by Piquet et al [9] that is,

$$\text{RDI}=\;\frac{\left({\text{P}}_{1}-70\right)+2\;({\text{P}}_{2}-{\text{ P}}_{0})}{10}$$

where P_0_ is 15 s average resting HR, in our case, from 3 min 45 s to 4 min, P_1_ is the maximum HR recorded during the first 15 s of recovery, and P_2_ is the 15 s average after the 1^st^ minute of recovery, that is from 1 min and 00 s and 1 min and 15 s.

### Statistical analysis

**Fig 1:**
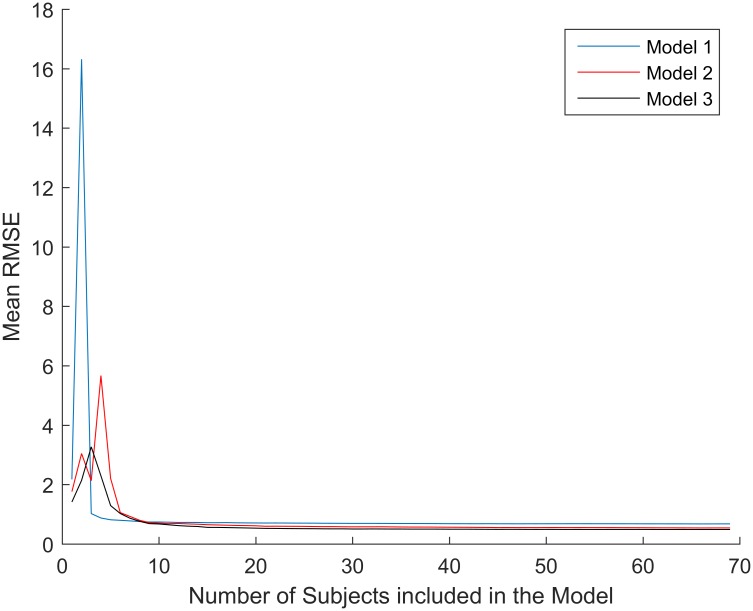
Features stability. These were assessed by calculating the grand average of the root mean squared error (RMSE) of each model 1, 2, and 3 as subject’s number included in the evaluation increased.

The statistical analysis was conducted in Matlab. The prediction models were built by using stepwise forward multiple linear regressions. In order to assess the repeatability of the ${\dot{\text{V}}}_{\text{O}2\text{max}}$ prediction using squat test HR derived features and models, 19 subjects performed a test re-test protocol from which we have calculated the Pearson correlation coefficient, and the intra-class coefficient (ICC), by using the following equation

$$\text{ICC}=\;\frac{\text{MSr}-\text{MSe}}{\text{MSr}}$$

where MSr is the mean square of the scores in test 1 and test 2, and MSe is the mean square error. The leave one out method was used to cross-validate the models. This means that the dataset was divided randomly into a number of groups equal to the number of subjects. Any iteration data among each subject was removed from the dataset and the model was tested against the remaining observations. Moreover, a simulation for evaluating the stability of the model features was performed. This was done by calculating the grand average of the root mean squared error (RMSE) of each model, developed in this study, as subject’s number increased (Fig 1). The RMSE was calculated from a random “testing” subset (10% of the full dataset) using a model trained on an increasing number of subjects (1 to 73, that is, full dataset—size of testing data set) belonging to a fictitious “training” dataset. Subjects were randomly included in this “training” dataset to remove any possible order effect. This procedure was repeated in 100 iterations (arbitrary number) per number of subjects included in the “training” dataset and the grand average RMSE was computed.

In order to assess the quality of the RDI and our newly developed models in classifying CRF levels against the American College of Sports Medicine (ACSM) norms based on the directly measured ${\dot{\text{V}}}_{\text{O}2\text{max}}$ [10], sensitivity and specificity were calculated as follows. According with the original RDI classification of CRF levels [8], we made 3 categories: RDIs ≤ 5 were considered good CRF, RDIs between 6 and 10 were considered fair CRF, and RDIs ≥ 11 were classified as poor CRF. Consistently, we have simplified the ACSM classification in the same 3 categories, pooling together very poor and poor into poor, and good excellent and superior into good. Sensitivity was defined as:

$$\text{Sensitivity}=\;\frac{\text{nTP}}{\text{nTP}+\text{nFN}}$$

where nTP was the number of true positive, which in our case was estimation models CRF level equal to ACSM CRF level for good and fair; and nFN was number of false negative, which was when the estimation models underestimated ACSM CRF levels. Specificity was calculated as:

$$\text{Specificity}=\;\frac{\text{nTN}}{\text{nTN}+\text{nFP}}$$

where nTN was the number of true negative, that is, when the models and the ACSM classifications agreed on the poor CRF levels; and nFP was the number of false positive, namely, when the models overestimated ACSM CRF levels. The false positive rate was computed as 1 –Specificity. Moreover, the agreement between the ACSM classification and the models classification was evaluated with Cohen’s kappa coefficient (κ).

## Results

**Fig 2:**
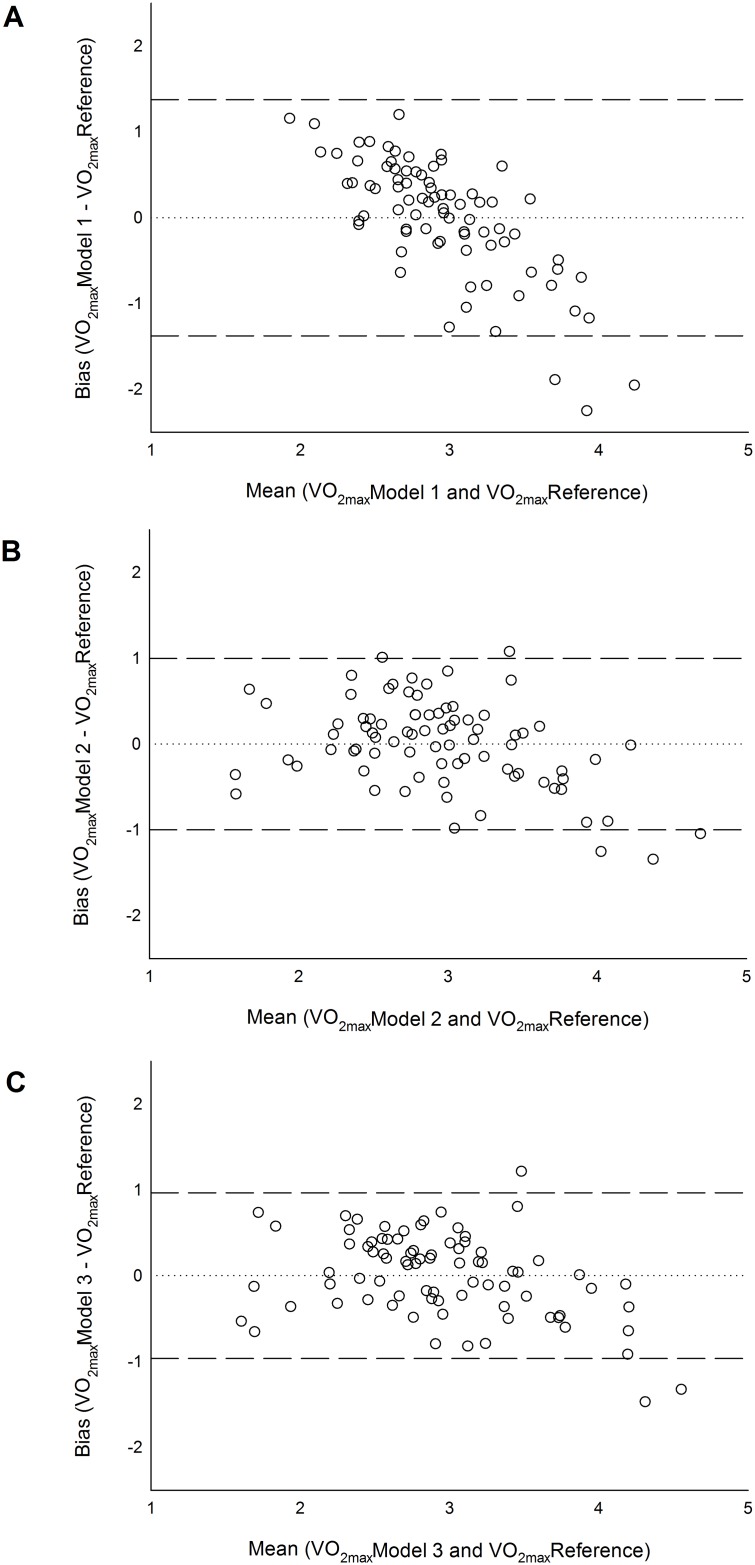
Bland and Altman plots. A, Model 1 based on RDI only; B, Model 2 based on RDI and subject’s characterises; C, Model 3 based and sex and Start HR_rec_ normalized for height and Age; n = 81.

In Table 2 we report the linear relations between subjects’ characteristics, the HR features at rest, during squatting and recovery and the absolute ${\dot{\text{V}}}_{\text{O}2\text{max}}$. Three multiple linear regression models to predict absolute ${\dot{\text{V}}}_{\text{O}2\text{max}}$ were developed. Model 1 and 2 were based on the RDI, whereas models 3 made used of HR recovery features developed in this study (Fig 2). The accuracy and cross-validation errors of these models are reported in Table 3.

**Table 2:** Pearson correlation coefficients for absolute ${\dot{\text{V}}}_{\text{O}2\text{max}}$ (L·min^-1^).

	Males	Females	All
	n = 63	n = 18	n = 81
Sex	-	-	0.480 (p<0.001)
Age	-0.463 (p<0.001)	-0.326	-0.325 (p = 0.003)
Height	0.331 (p = 0.008)	0.552 (p = 0.017)	0.483 (p<0.001)
Weight	-0.046	0.350	0.143
Rest HR mode	-0.169	-0.400	-0.262 (p = 0.018)
HR max (during ${\dot{\text{V}}}_{\text{O}2\text{max}}$ test)	0.521 (p<0.001)	0.300	0.459 (p<0.001)
Squat HR peak	-0.256 (p = 0.043)	0.096	-0.279 (p = 0.011)
Start HR_rec_	-0.381 (p = 0.002)	-0.123	-0.374 (p<0.001)
Recovery exponential coefficient	-0.319 (p = 0.011)	0.094	-0.291 (p = 0.008)
Recovery HR end	-0.286 (p = 0.023)	-0.531 (p = 0.023)	-0.339 (p = 0.002)
RDI	-0.322 (p = 0.010)	-0.554 (p = 0.017)	-0.402 (p<0.001)
Start HR_rec_ / Age^2^	0.517 (p<0.001)	0.289	0.238 (p = 0.032)
Start HR_rec_ / H^2^	-0.505 (p<0.001)	-0.354	-0.549 (p<0.001)

Start HR_rec_ = Recovery HR linear intercept, H = Height

**Table 3:** Multiple linear regression models to predict ${\dot{\text{V}}}_{\text{O}2\text{max}}$ (n = 81).

	Coef.	SE	t	*p* level	r	Adj.r^2^	RMSE	Bias	LoA	LOOCV
							(L·min^-1^)	(L·min^-1^)	(L·min^-1^)	(L·min^-1^)
**Model 1**					0.402	0.151	0.707	-0.001	1.375	0.676
									-1.377	
Constant	3.867	0.247	15.650	<0.001						
RDI	-0.110	0.0281	-3.905	<0.001						
**Model 2**					0.747	0.535	0.523	0.001	0.999	0.532
									-0.998	
Constant	-3.788	1.798	-2.107	0.038						
Sex	0.560	0.159	3.534	<0.001						
Age	-0.0309	0.007	-4.393	<0.001						
Height	4.533	1.051	4.314	<0.001						
RDI	-0.0864	0.021	-4.014	<0.001						
**Model 3**					0.766	0.587	0.502	0.001	0.967	0.495
									-0.965	
Constant	4.121	0.458	9.007	<0.001						
Sex	0.787	0.147	5.357	<0.001						
Start HR_rec_/Age^2^	4.235	0.708	5.979	<0.001						
Start HR_rec_/H^2^	-0.0673	0.011	-6279	<0.001						

SE = Standard error, RMSE = root mean squared error, LoA = limits of agreement, LOOCV = leave one out cross validation error, RDI = Ruffier-Dickson index, H = height

Nineteen individuals had repeated the squat test a second time on a different day at least one week after the first test. Intra-class correlation coefficient for Squat HR peak was 0.86 (r’ = 0.76, p<0.001), ICC for Start HR rec was 0.79 (r’ = 0.66, p<0.01) and the ICC for RDI was 0.82 (r’ = 0.70, p<0.001). Intraclass correlation coefficient for model 1 was the same as for RDI, being this model based solely on RDI, ICC for model 2 was 0.91 (r’ = 0.83, p<0.001), finally ICC for model 3 was 0.93 (r’ = 0.88, p<0.001).

As described in the methods we have made 3 fitness classes based on the ACSM norms, poor, average and good. Out of our 81 subjects 40 belonged to the poor fitness category, 24 to the average fitness and 17 to the good fitness. Model 1 has shown a sensitivity to classify CRF levels according with the ACSM norms equal to 61%, whereas its specificity was 49%, meaning that the rate of false positive (e.g. subjects wrongly classified as fit) was 51%. There was a fair agreement between model 1 and ACSM CRF levels (κ = 0.293). Model 1 misclassified 7 individuals over 81 (9%) by two categorises.

Model 2 showed a better sensitivity 62% and specificity 63%, reducing the miss classification of poor to average into fit to 37%. Model 2 also had a fair agreement with the ACSM classification of CRF (κ = 0.397). Model 3, similarly to model 2, had 64% sensitivity and 62% specificity, with a moderate agreement with the classification of CRF as according with the ACSM norms (κ = 0.417). Model 2 and 3 improved on two categories misclassification, dropping this down to 1 over 81 observations (1%).

In order to assess the aerobic requirements of the squatting exercise breath by breath O_2_ uptake measurements were performed during the squatting protocol on 10 subjects. This revealed a mean peak metabolic equivalent (MET) of 6.27 ± 2.23. One MET was defined as an O_2_ uptake of 3.5 ml ∙ kg^-1^. According to these results this test can be classified as aerobically vigorous exercise in the less fit and moderate exercise in fitter individuals.

## Discussion

${\dot{\text{V}}}_{\text{O}2\text{max}}$ can be estimated by executing a 45 s squat test in healthy individuals of both sexes. The RDI, which uses both resting and recovery HR information, significantly correlated with absolute ${\dot{\text{V}}}_{\text{O}2\text{max}}$. However, RDI was not the best HR metric to estimate CRF or ${\dot{\text{V}}}_{\text{O}2\text{max}}$. The best HR feature in the current study was the intercept of the linear fit of the first 180 s of recovery, (called here start HR_rec_).

The oxygen uptake measured while performing 30 squats in 45 seconds has verified the moderate to vigorous impact (~6 peak MET) of this test on the aerobic system and its associated cardiorespiratory load (128 beats · min^-1^ peak HR). We expected individuals’ height to play a key role in influencing the hemodynamic responses induced by repetitive squatting. This is because the displacement of the centre of mass is larger in taller individuals, corresponding to a greater exercise dose which elicits a greater cardiac work [11]. In fact once we accounted for height differences, dividing start HR_rec_ by height squared, the differences in HR were mainly dependent on CRF levels and less on anthropometric factors. Indeed, we observed a higher correlation with absolute ${\dot{\text{V}}}_{\text{O}2\text{max}}$ when using start HR_rec_/H^2^. Similarly, we have corrected the start HR_rec_ for age, which is known to negatively relate with ${\dot{\text{V}}}_{\text{O}2\text{max}}$ [12]; also this correction increases the correlation with CRF. Interestingly, body weight did not show an important correlation with absolute ${\dot{\text{V}}}_{\text{O}2\text{max}}$. This is not surprising considering that body weight alone does not necessarily reflect fat free mass [13] and that ${\dot{\text{V}}}_{\text{O}2\text{max}}$ measured on the cycle ergometer does not scale with body weight [14]. In a previous investigation we have developed a prediction equation based on squatting data gathered in 22 male rugby players by Piquet et al [9], using body weight, age and, end of squatting exercise HR as prediction variables; on that limited dataset a high correlation was observed (r = 0.75) [1]. However, when that equation was tested in this current dataset no correlation with ${\dot{\text{V}}}_{\text{O}2\text{max}}$ (r = -0.0089) was found. This confirmed that body weight is not a good predictor of fitness for this type of submaximal test and that end exercise HR by itself is not a good predictor of CRF.

The Ruffier-Dickson index takes into account resting as well as recovery HR parameters. In particular it takes into account start recovery HR, which is very close to peak values, and the difference between 1 minute recovery HR and resting HR. Table 2 shows how these HR parameters well correlate with CRF. Once the RDI was entered in a linear regression model (Model 1) it was observed that although the mean bias was very close to zero, this model had the tendency to overestimate CRF in low fitness level people and conversely underestimate CRF in fit people (Fig 2A). This was statistically confirmed by a significant negative heteroscedasticity. This drawback was mitigated by including in the regression model information about sex, age and height (Model 2). Model 2, RDI plus individual’s characteristics, also improved on accuracy dropping the cross-validation estimation error from 23% down to 18%. If no advance HR features extraction tools were available, Model 2 should be use to estimate CRF when using the 45 s squat protocol described in this study. This is because individual’s characteristics improve in personalization and therefore in prediction and reduce the risk of fitness bias.

However, the best multiple linear regression model was based on HR recovery curve linear fit normalized as described above. This third model had only three predictive variables, sex start HR_rec_/H^2^, start HR_rec_/Age^2^. Model 3 showed the lowest cross validation error 16.8%, the best repeatability (ICC = 0.93), and the best CRF classification performance. It is important to notice that, although a moderate agreement (κ = 0.417) between gold standard and Model 3 in classifying CRF in 3 categories, poor, average and good, may seem low, the κ coefficient, in fact, accounts for random agreements. Indeed, a κ equal to 0 would be an agreement purely by chance [15]. Moreover, it also relevant to report that Model 3 as well as Model 2 had a very low two categories misclassification rate (1%). This analysis, which is rarely done when validating CRF tests, was very important when considering that the RDI was originally conceived to classify people according to their fitness level [8]. The RDI alone was observed to have poor specificity, meaning that the rate of people placed in a higher fitness level category was very high (51%). Thus, the RDI should not be used alone for CRF classifications. We have also evaluated how the sample size could affect the accuracy of our models. In Fig 1, it is evident that the feature selected show stable and rather low errors when more than 10 random individuals are included in the models. This simulation together with the leave one out cross-validation gives us confidence that the accuracy shown in this dataset will be reproducible in other data. The error shown by model 3 (16.8%) is on par with well-known submaximal protocols such as the Rockport walk test on the treadmill (15%) [16], or the ACSM cycling test (15.5%) [17]. Moreover, model 3 proved to be as repeatable as most submaximal protocols (ICC = 0.93, and r’ = 0.88), e.g. the Queens’ College step test (r’ = 0.92) [18], or the ACSM cycling test (r’ = 0.86) [17].

This study has strengths and limitations. The main strength is that it provides an easy-to-use test (e.g. Model 2) to evaluate CRF in healthy people with a wide range of fitness levels. The main limitation of the current work is that only healthy people not taking heart rhythm medications were included. It is logical to assume that HR-based CRF estimations would not be appropriate in heart patients taking such medications. A lower HR value would be read as higher fitness by our prediction models instead of higher medication dose. In order to address this issue, we have elsewhere disclosed and validated a novel approach, which makes use of the same squatting protocol, but based on body movements [19]. Moreover, a 45 second protocol was used to replicate the original Ruffier-Dickson test. However, this may not have been the optimal duration for assessing CRF by means of squatting. Future, research should investigate the optimal duration for this squatting protocol.

In conclusion, a simple 45 s squat test can be used to estimate CRF or ${\dot{\text{V}}}_{\text{O}2\text{max}}$ in healthy males and females with moderate accuracy and large repeatability when HR recovery features of the first 180 s are used. Furthermore, our newly developed model (model 3) can classify CRF levels reasonably well. Although the RDI alone significantly correlates with the absolute ${\dot{\text{V}}}_{\text{O}2\text{max}}$ and its repeatability was acceptable, it seems to overestimate ${\dot{\text{V}}}_{\text{O}2\text{max}}$ for individuals with low ${\dot{\text{V}}}_{\text{O}2\text{max}}$ and underestimate it in individuals with high ${\dot{\text{V}}}_{\text{O}2\text{max}}$. RDI performance was improved when individual’s characteristics, such as sex, age, height were added to the model.
